# MicroRNAs as potential biopredictors for premenopausal osteoporosis: a biochemical and molecular study

**DOI:** 10.1186/s12905-023-02626-3

**Published:** 2023-09-09

**Authors:** Hadeel A. Al-Rawaf, Sami A. Gabr, Amir Iqbal, Ahmad H. Alghadir

**Affiliations:** 1 Department of Clinical Laboratory Sciences, College of Applied Medical Sciences, King Saud University, Riyadh, 11433 Saudi Arabia; 2 Department of Rehabilitation Sciences, College of Applied Medical Sciences, King Saud University, Riyadh, 11433 Saudi Arabia

**Keywords:** MicroRNAs, Osteoporosis, BMD, QUS, c-BUA, Serum bone markers

## Abstract

**Background:**

Circulating micro-RNAs have been proposed as a new type of biomarker in several diseases, particularly those related to bone health. They have shown great potential due to their feasibility and simplicity of measurement in all body fluids, especially urine, plasma, and serum.

**Aim:**

This study aimed to evaluate the expression of a set of mRNAs, namely miR-21, miR-24, mir-100, miR-24a, miR-103-3p, and miR-142-3p. Their proposed roles in the progression of osteoporosis were identified using a real-time polymerase chain reaction (RT-PCR) analysis in premenopausal women. In addition, their correlations with osteocalcin (OC), bone-specific alkaline phosphatase (BAP), and deoxypyridinoline (DPD) bone markers were explored.

**Methods:**

A total of 85 healthy premenopausal women aged 25–50 years old were included in this study. Based on a DXA scan (Z-score) analysis and calcaneus broadband ultrasound attenuation scores (c-BUAs), measured via quantitative ultrasound (QUS), the subjects were classified into three groups: normal group (n = 25), osteopenia (n = 30), and osteoporosis (n = 30). Real-time-PCR and immunoassay analyses were performed to determine miRNA expression levels and serum OC, s-BAP, and DPD, respectively, as biomarkers of bone health.

**Results:**

Among the identified miRNAs, only miR-21, miR-24, and mir-100 were significantly upregulated and increased in the serum of patients with osteopenia and osteoporosis, and miR-24a, miR-103-3p, and miR-142-3p were downregulated and significantly decreased in osteoporosis. Both upregulated and downregulated miRNAs were significantly correlated with BMD, c-BUA, OC, s-BAP, and DPD.

**Conclusion:**

A group of circulating miRNAs was shown to be closely correlated with the parameters BMD, c-BUA, OC, s-BAP, and DPD, which are traditionally used for bone-health measurements. They could be identified as non-invasive biomarkers in premenopausal patients with osteoporosis. More studies with large sample sizes are recommended to estimate the mechanistic role of miRNAs in osteoporosis pathogenesis and to provide evidence for the use of these miRNAs as a non-invasive method of diagnosing clinical osteoporosis, especially in premenopausal patients.

## Introduction

Bone loss has been recognized as a global health problem [1], and there have been increasing trends of low bone mass. Conditions such as osteoporosis and osteopenia, have been reported to be associated with bone fracture risk [2, 3], age, and many basic metabolic disorders, such as dyslipidemia and obesity. In addition, poor lifestyle factors, such as irregular nutrition, hormonal status, toxin exposure, medications, lower physical activity, and educational levels, are significantly associated with higher ratios of bone mineral density (BMD) loss [4–9]. Low bone mineral density (BMD) mainly results from increased bone resorption, performed by osteoclasts, compared with bone formation, performed by osteoblasts [10].

Several biochemical markers such as deoxypyridinoline (DPD), related to the bone resorption process, and both osteocalcin (OC) and bone-specific alkaline phosphatase (BAP), related to bone formation, have been validated as useful markers of bone metabolism, bone loss, and osteoporosis [11–15]. Their changes are easily estimated in the urine, serum, or plasma. They have been significantly linked to the diagnosis of bone loss or osteoporosis with validated tools that are used in clinical routine such as X-ray- and ultrasound-based techniques, especially DEXA scans and quantitative ultrasounds (QUS) [16–20].

Although these validated biomarkers have been assessed in bone diseases via DXA [21], especially as early markers of drug efficacy, they still have some specific limitations. These include a lack of normative reference in population databases, poor standardized quality control, and poor sample handling [21], as well as having only a moderate association with bone-strength and fracture-risk conditions [22, 23]. Thus, new novel biomarkers should be evaluated to better diagnose bone diseases such as osteoporosis, strength, and fracture risks in patients.

Recently, circulating microRNAs have been reported to be associated with many bone diseases [24]. They are short non-coding RNAs, which are reported to have regulatory roles in gene expression in most cells and tissues, including muscle and bone [25–27], usually by suppressing translation or destabilizing mRNAs [28]. Previous research studies have reported the involvement of mRNAs in the process of osteoblastogenesis [29–31]. Recently, the upregulation of 49 miRNAs and downregulation of 44 miRNAs were estimated, respectively, during the early to late differentiation stages of osteoclastogenesis [32–34].

However, no studies refer to the correlation between miRNAs as biomarkers of bone diseases and previously validated bone markers, as well as routinely used X-ray- and ultrasound-based techniques. Although molecular markers are more expensive than the usual bone-identification markers, we hypothesize in this study that miRNAs, as biomarkers of bone loss, might correlate with previously reported bone markers as well as X-ray- and ultrasound-based techniques to provide new molecular insights that could aid in the treatment of bone loss.

Thus, this study aimed to evaluate the expression of a set of mRNAs, namely miR-21, miR-24, mir-100, miR-24a, miR-103-3p, and miR-142-3p, and their proposed roles in the progression of osteoporosis were identified via q-RT-PCR analysis in premenopausal women. In addition, their correlations with OC, sBAP, and DPD bone markers were explored.

## Materials and methods

### Subjects

A total of 85 healthy, non-smoking, premenopausal women aged 25–50 years old were included in this study. Patients with mental or physical functional impairments, hormonal disorders, secondary osteoporosis, liver or kidney diseases, or those taking medications such as antipsychotics, oral anticoagulants, corticoids, calcium, and vitamin D supplements that interfere with bone turnover were excluded from this study. Based on the clinical features and diagnosis evidence from both the DXA scan (Z-score) and quantitative ultrasound (QUS) analysis, subjects were classified into three groups: Group A (control subjects with normal BMD; Z-score; ˃ -2.5; n = 25); Group B (premenopausal patients with low BMD; Z-score; -1 SD to -2.5 SD; n = 30); Group C (premenopausal subjects with low BMD; Z-score; ≤ − 2.5; n = 30). For all participants, standard anthropometric measurements, i.e., BMI, WHR, and WC, were estimated as previously reported [48].

Regarding the ethical guidelines of the 1975 Declaration of Helsinki, the study protocol was reviewed and approved by the Ethics Sub-Committee of the King Saud University, Kingdom of Saudi Arabia, under file number ID: RRC-2016-049. All participants signed a written informed consent document before data collection. Blood was collected from all subjects, and serum samples were obtained following centrifugation for 1 min at 1400 rpm. The samples were given a coded study identification number and were shipped frozen at 20° C for analysis. The demographic and clinical data of the participants are shown in Table [1].

### Assessment of bone mineral density (BMD)

The BMD (g/cm^2)^ of the lumbar spine (L2-L4) and proximal femur of the participants was estimated using the Dual Energy X-ray Absorptiometry (DEXA, UNIGAMMA PLUS AC 230 V 50/60Hz 400w, USA) scan method. Osteoporosis was diagnosed among participants using the DEXA method according to their Z-score: Group A (control subjects with normal BMD; Z-score; ˃ -2.5; n = 25); Group B (premenopausal patients with mild low BMD; Z-score; -1 SD to -2.5 SD; n = 30); Group C (premenopausal subjects with moderate low BMD; Z-score; ≤ − 2.5; n = 30) [35].

### Calcaneus Broadband Ultrasound attenuation test (C-BUA)

As mentioned previously, a commercially available Achilles ultrasound densitometer (Lunar Corporation, Madison, WI, USA) was used to estimate bone health among all participants [36, 37]. This technique was used briefly to measure the c-BUA values in patients with osteoporosis [16–19]. As suggested in clinical practice, patients with poor bone health are those with a Z-score cut-off of ≤ -1.5 SD [38], and the coefficient of variation (CV) for c-BUA values ranges from 0.69 to 1.8% during the day of measurement, as mentioned in the literature [39].

### Assessment of bone markers

Enzyme immunoassay kits (Metra Biosystems, Mountain View, CA, USA) were used to estimate the levels of DPD as a bone resorption marker in the urine samples of participating patients. Based on the urinary creatinine concentration, the DPD values of all subjects were normalized and expressed as nmol DPD/mmol creatinine. The patients were classified according to the grading scores of DPD into the following categories: normal BMD (DPD; 3.4–7.4), low BMD loss (DPD; 7.4–15), higher BMD loss (DPD; 16–30), and very high BMD loss (DPD; >30), whereas subjects with higher DPD values were supposed to have osteoporosis, as previously reported [40]. In addition, both serum BAP (sBAP) and osteocalcin (OC) were measured in the serum samples of patients using an enzyme immunoassay kit (Alka phase, Metra Biosystems) for sBAP and the MicroVue Osteocalcin enzyme immunoassay (QUIDEL Corporation, San Diego, CA) for serum OC, respectively [35, 41].

### Isolation of miRNAs and RT-PCR

For each subject, the total RNA was extracted from serum samples using the TRIzol LS reagent (Invitrogen, Carlsbad, CA), and subjected to RT-PCR analysis. A ready-made solution containing the primers and probes for human miR-34a, miR-142-3p, miR-103-3p, miR-21, miR-24, and miR-100 (Applied Biosystems, Foster City, CA), and real-time RT-PCR, was estimated using an ABI 7300 system (Applied Biosystems) [42]. RNU43 was used as an endogenous reference control, and all PCR cycles were performed according to the manufacturer’s instructions as previously described [43], whereas the relative quantification of miRNAs was performed using the 2 − ΔCt method. To avoid errors and to precisely determine the cycle threshold mean values for each sample, including amplified miRNAs and the endogenous control, all reactions were run in duplicate.

### Statistical analyses

The power calculations of the selected sample size of 85 subjects gave an estimated power of 95% and a significance level of 0.05 with an expected frequency of 8.6%.

The Shapiro–Wilk test was performed for normal data distribution and was logarithmically subjected to statistical analyses. To measure the differences between the studied groups of subjects, both Student’s t-test and ANOVA, followed by Bonferroni’s multiple comparison analysis, were used. The microRNA levels were adjusted for comparison via univariate analysis, using a general linear model for the different groups. Multiple stepwise regressions and Pearson’s correlation analysis were used to estimate the associations between microRNA levels, obesity, and OC, DPD, and sBAP bone parameters.

## Results

A total of 85 premenopausal patients were recruited in this study. Based on clinical features and diagnosis evidence from both the DXA scan (Z-score) and quantitative ultrasound (QUS) analysis, about 29.4% of the study subjects (n = 25) showed normal bone health (DXA Z-score; ˃ -1; c-BUA; Z-score; > -1.5; n = 25), and 70.4% of the study population showed abnormal bone health with a low BMD; they were classified into two groups: premenopausal patients with mild low BMD (group B; DXA Z-score; -1 SD to -2.5 SD; c-BUA; Z-score ≤ -1.5; n = 30) and premenopausal subjects with moderate low BMD ( group C; DXA Z-score; ≤–2.5; c-BUA; Z-score ≤ -1.5; n = 30), as shown in Table [1]. BMI and WHR, as markers of obesity, showed a significant increase in patients with osteopenia and osteoporosis compared to healthy controls.

Also, the values of c-BUA, SOS, and BMD outcome measures for bone health showed a significant decrease in patients with mild (P < 0.01) and moderate (P < 0.001) low BMD, respectively, compared to controls with normal BMD. Conversely, OC, sBAP, and DPD as serum markers of bone metabolism showed a significant increase in both patients with mild BMD (P < 0.01) and moderate BMD (P < 0.001) [Table 1]. BMD, c-BUA, and SOS, as outcome variables of bone health, were significantly correlated with obesity and markers of bone metabolism. They correlated negatively with obesity (BMI; P < 0.001) and positively (P < 0.001) with OC, s-BAP, and DPD in patients with mild (P < 0.01) and moderate BMD, respectively, as shown in Table 2.

**Table 1:** Demographic and clinical data of the participants (N = 85)

Parameters		Group (A) Controls (n = 25; 29.4%)	Group (B) Premenopausal with mild low BMD (n = 30; 35.3%)	Group (C) Premenopausal with moderate low BMD (n = 30; 35.3%)
Age (years)		43.4 ± 2.7	43.8 ± 1.9	43.5 ± 2.6
BMI (kg/m2)		24.5 ± 1.6	26.2 ± 3.8 ^a^	26.7 ± 2.7 ^a^
WHR		0.78 ± 0.38	0.89 ± 0.68 ^a^	0.92 ± 0.81^a^
Waist (cm)		79.3 ± 10.5	86.4 ± 6.5^a^	91.4 ± 8.4
TBMD		1.4 ± 0.98	0.98 ± 0.86 ^a^	0.89 ± 0.058^b^
BMD (Z Score)	Lumbar spine	1.5 ± 0.75	-1.85 ± 0.85 ^a^	-2.15 ± 1.2 ^a^
	Femoral neck	1.2 ± 1.3	-1.89 ± 0.8 ^a^	-2.089 ± 0.5 ^a^
c-BUA (dB/MHz)		59.6 ± 2.3	48.1 ± 1.4^a^	39.5 ± 1.2 ^b^
SOS (m/s)		1645.2 ± 4.5	1638.4 ± 3.1^a^	1635.9 ± 18.1 ^b^
OC (ng/ml)		12.7 ± 3.2	18.3 ± 2.5^a^	26.1 ± 4.7 ^b^
DPD (nmol/mmol creatinine)		5.48 + 1.05	6.8 + 3.8 ^a^	12.3 + 1.78 ^b^
sBAP (U/l)		14.8 ± 3.1	16.9 ± 4.2 ^a^	21.2 ± 3.8 ^b^

Data expressed as mean ± SD, median (interquartile range), standard deviation (SD); body mass index (BMI); waist-to-hip ratio (WHR); bone mineral density (BMD); broadband ultrasound attenuation (BUA); speed of sound (SOS); serum bone alkaline phosphatase (s-BAP). DPD: deoxypyridinoline; osteocalcin level (ng/ml). ^a^ P < 0.05; ^b^ P < 0.01

**Table 2:** Association of BMD and c-BUA as outcome variables of bone health with adiposity, serum levels of bone resorption, and formation markers in premenopausal patients with bone loss (varying ranges of low BMD) (n = 60; 70.6%)

Parameters	Bone-health status {BMD scores^(c)^, c-BUA^(d)^ }					
	Control		Mild low BMD		Moderate Low BMD	
	R^2^ (β) ^a^	95% CI	R^2^ (β) ^b^	95% CI	R^2^ (β)^b^	95% CI
BMI	-2.3(0.21)	91(89–96)	-3.7(0.12)	94(89–100)	-3.8(0.28)	89(75–96)
OC	4.2(0.15)	86(78–97)	5.8(0.31)	94(88–100)	5.8 (0.58)	91(88–100)
DPD	5.1(0.17)	95(87–100)	7.5(0.42)	96(89–100)	9.4(0.68)	94(89–100)
sBAP	3.7(0.32)	90(86–98)	6.8(0.48)	89(84–100)	7.5(0.75)	86 (78–100)
OC	2.7(0.38)	89(78–97)	8.7(0.56)	96(86–100)	10.1(0.86)	96 (87–100)

Beta coefficient (β) and cumulative *R*^2^* derived from the stepwise regression analysis model showed additional significant variables that were added to the model via bivariate analysis. BMI: body mass index; OC: osteocalcin; sBAP: bone alkaline phosphatase; BMD: bone mineral density; DPD: deoxypyridinoline; SOS: speed of sound; c-BUA: calcaneus quantitative ultrasound parameter. (c) BMD Z-score; Group A (control subjects with normal BMD; Z-score; ˃ -2.5; n = 25); Group B (premenopausal patients with mild low BMD; Z-score; -1 SD to -2.5 SD; n = 30); Group C (premenopausal subjects with moderate low BMD; Z-score; ≤ − 2.5; n = 30); (d) c-BUA; Z-score; normal or healthy bone (> -1.5); poor bone or unhealthy bone (Z-score ≤ -1.5). ΣR2 = summation of cumulative values of R relating to studied variables. *P < 0.01; **P < 0.001

**Fig. 1:**
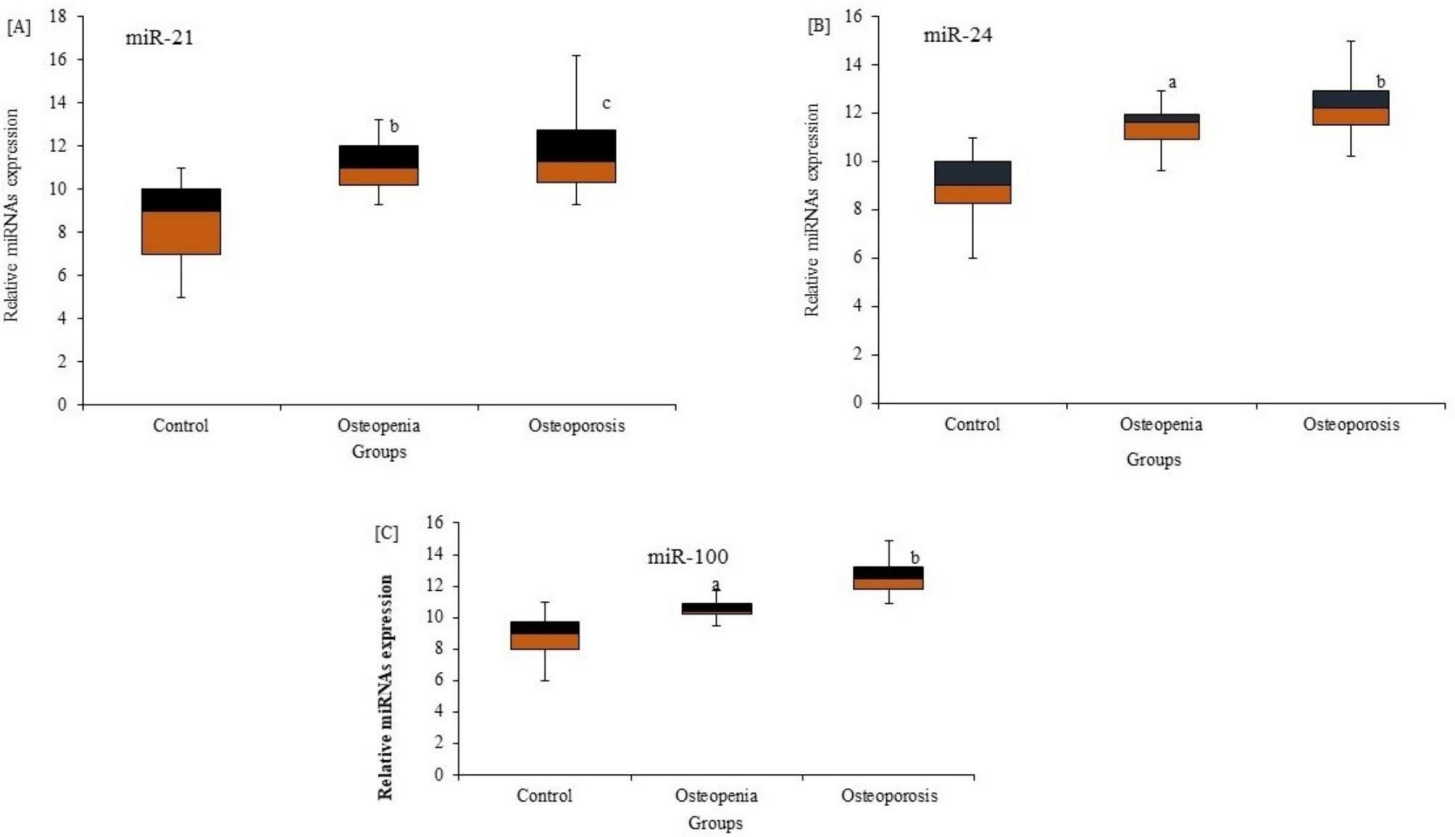
Differential expression of serum miRNAs in premenopausal females with varying degrees of bone loss (L-BMD) compared to healthy controls. The expression of miRNA-21 (**A**), miRNA‐24 (**B**), and miRNA‐100 (**C**) was significantly increased (upregulated) in female patients with different bone-loss scores indicated by lower BMD, mild L-BMD (P = 0.01), and moderate L-BMD (p = 0.001) compared to healthy control subjects. ^a^ p < 0.05, ^b^ p < 0.01, ^c^ p < 0.001 for the comparison indicated by Mann–Whitney U test. Group A: healthy subjects with normal BMD; Group B: premenopausal subjects with mild L-BMD; Group C: premenopausal subjects with moderate L-BMD

**Fig. 2:**
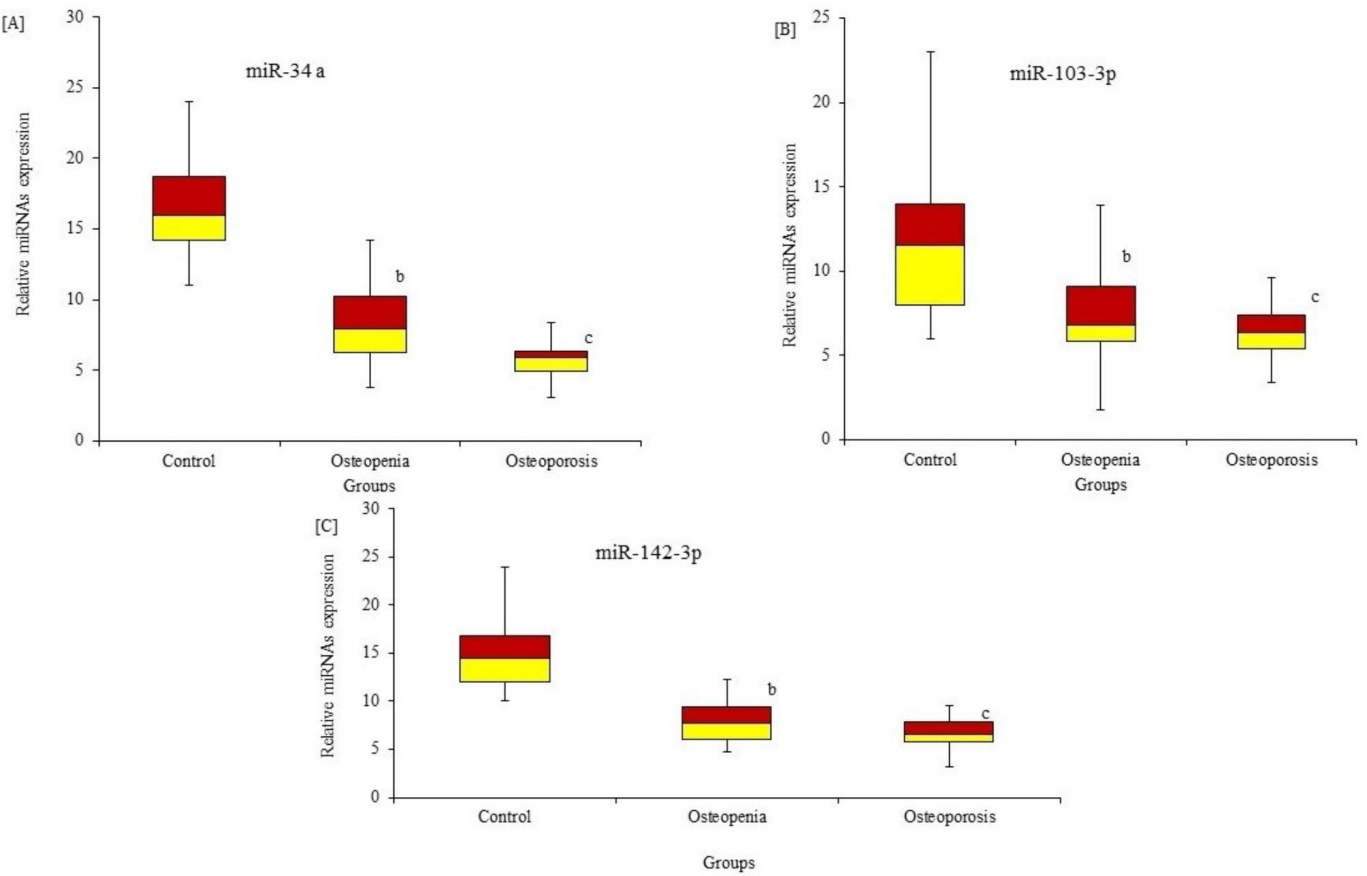
Differential expression of serum miRNAs in premenopausal females with varying degrees of bone loss (L-BMD) compared to healthy controls. The expressions of miRNA-34 a (**A**), miRNA‐103-3p (**B**), and miRNA‐142-3p (**C**) were significantly reduced (downregulated) in the serum of premenopausal females with mild (P = 0.01) and moderate (p = 0.001) bone loss (L-BMD) compared to healthy control subjects. ^* a^ p < 0.05, ^b^ p < 0.01, ^c^ p < 0.001 for the comparison indicated by Mann–Whitney U test. Group B: premenopausal subjects with mild L-BMD; Group C: premenopausal subjects with moderate L-BMD

MicroRNAs as biomarkers for bone health were estimated in all subjects (Fig. 1). A set of miRNAs, including miR-21, miR-24, mir-100, miR-24 a, miR-103-3p, and miR-142-3p, was estimated in all subjects via quantitative RT-PCR analysis. MiR-21, miR-24, and miR-100 were significantly (P < 0.001) upregulated and highly expressed in patients with mild and moderate low BMD compared to control subjects with normal BMD [Fig. 1A, B & C]. Conversely, miR-24 a, miR-103-3p, and miR-142-3p were significantly downregulated and reduced in their expression among patients with mild and moderate low BMD compared to respective control subjects with normal BMD [Fig. 2A, B &C]. The results showed that the varied expression of different microRNAs could have a pathogenic role in the degree of bone loss measured by variations in low BMD.

Correlation coefficient analysis showed that both the upregulation and downregulation of the expressed miRNAs were significantly correlated with the serum levels of OC, s-BAP, and DPD as biomarkers of bone metabolism [Table 3]. The bone metabolism markers, c-BUA, and BMD outcome measures for bone health correlated positively with the upregulated miRNAs, miR-21, miR-24, mir-100, and negatively with the downregulated miRNAs, miR-24 a, miR-103-3p, and miR-142-3p, as shown in Table [3] and Table [4].

**Table 3:** Correlation analysis of miRNA expression levels with bone formation and resorption markers in premenopausal patients with varying ranges of low BMD (n = 60; 70.6%)

Parameters	OC		sBAP		DPD	
**Mild low BMD (n = 30)**	R^2^ (β) ^a^	95% CI	R^2^ (β)^b^	95% CI	R^2^ (β)^b^	95% CI
Upregulated miRNAs						
miR-21	2.3(0.19)	89 (78–96)	3.5(0.59)	92 (88–96)	7.1(0.65)	91 (84–98)
miR-24	3.6(0.16)	92 (86–98)	5.1(0.35)	97 (87–100)	4.8(0.48)	95 (89–98)
miR-100	4.6(0.27)	85 (75–92)	8.4(0.58)	84 (78–94)	9.1(0.68)	89 (78–96)
Downregulated miRNAs						
miR-34 a	7.2(0.32)	75 (71–89)	8.1(0.65)	76 (81–92)	6.4(0.75)	78 (79–95)
miR-103-3p	6.4(0.28)	96 (85–98)	8.9(0.34)	90 (88–100)	7.1(0.81)	93 (88–100)
miR-142-3p	8.1(0.52)	90 (81–96)	6.2(0.57)	93 (85–98)	5.1(0.49)	96 (85–98)
**Moderate low BMD (n = 30)**						
Upregulated miRNAs						
miR-21	4.9(0.45)	86 (78–96)	4.7(0.71)	96 (89–100)	5.3(0.81)	95 (86–98)
miR-24	7.5(0.36)	94 (86–98)	8.3(0.78)	86 (78–96)	6.8(0.56)	93 (87–98)
miR-100	5.3(0.34)	81 (75–92)	5.2(0.65)	81 (78–94)	7.1(0.78)	91 (84–96)
Downregulated miRNAs						
miR-34 a	6.2(0.46)	88 (71–96)	9.3(0.78)	78 (81–96)	8.9(0.82)	91 (87–96)
miR-103-3p	3.1(0.31)	91 (88–97)	5.2(0.43)	95 (85–100)	7.5(0.86)	96 (89–100)
miR-142-3p	5.6(0.58)	92 (85–95)	7.2(0.63)	97 (85–100)	8.3(0.67)	90 (86–98)

Beta coefficient (β) and cumulative R2* derived from the stepwise regression analysis model showed additional significant variables added to the model via bivariate analysis. OC: osteocalcin; sBAP: bone alkaline phosphatase; DPD: deoxypyridinoline. ^a^P<0.01; ^b^P<0.001

**Table 4:** Association of BMD and c-BUA as outcome variables of bone health with miRNA expression levels in premenopausal patients with varying ranges of low BMD (n = 60; 70.6%)

Parameters	{BMD scores ^(c)^}		{c-BUA ^(d)^}	
**Mild low BMD (n = 30)**	R^2^ (β) ^a^	95% CI	R^2^ (β)^b^	95% CI
Upregulated miRNAs				
miR-21	4.1(0.12)	86 (80–96)	4.8(0.23)	89 (86–96)
miR-24	5.6(0.21)	94 (88–98)	6.1(0.17)	91 (82–100)
miR-100	4.9(0.32)	89 (73–99)	5.7(0.28)	94 (78–99)
Downregulated miRNAs				
miR-34 a	-5.1(0.32)	78 (68–91)	-7.3(0.34)	84 (79–98)
miR-103-3p	-3.8(0.31)	96 (81–98)	-4.8(0.36)	92 (88–100)
miR-142-3p	-6.7(0.58)	99 (87–100)	-6.9(0.38)	94 (85–100)
**Moderate low BMD (n = 30)**				
Upregulated miRNAs				
miR-21	6.2(0.25)	87 (76–96)	4.7(0.71)	96 (89–100)
miR-24	5.4(0.51)	92 (86–98)	8.3(0.78)	86 (78–96)
miR-100	5.8(0.38)	86 (78–96)	5.2(0.65)	81 (78–94)
Downregulated miRNAs				
miR-34 a	-8.1(0.58)	94(78–100)	-10.1(0.84)	91 (88–98)
miR-103-3p	-6.4(0.71)	89 (75–98)	-8.5(0.91)	89 (81–100)
miR-142-3p	-7.3(0.91)	90 (88–100)	-6.7(0.65)	95 (85–100)

Beta coefficient (β) and cumulative R2* derived from the stepwise regression analysis model showed additional significant variables added to the model via bivariate analysis. BMD: bone mineral density; c-BUA: calcaneus quantitative ultrasound parameter. (c) BMD Z-score; Group A (control subjects with normal BMD; Z-score; ˃ -2.5; n = 25); Group B (premenopausal patients with mild low BMD; Z-score; -1 SD to -2.5 SD; n = 30); Group C (premenopausal subjects with moderate low BMD; Z-score; ≤ − 2.5; n = 30); (d) c-BUA; Z-score; normal or healthy bone (> -1.5); poor bone or unhealthy bone (Z-score ≤ -1.5).^a^P<0.01; ^b^P<0.001

## Discussion

Bone health is controlled by a homeostatic dynamic equilibrium between the bone formation and bone resorption processes, which are mediated by osteoblasts and osteoclasts [44, 45]. Any changes or imbalances that occur in this equilibrium result in bone mass reduction, microarchitecture deterioration with an increased probability of bone fragility, and fracture risk [46]. Osteoporosis and osteopenia, as increasing trends related to low bone mass, were significantly reported in association with the probable risks of bone fracture [2, 3].

Bone loss, as measured using BMD and a c-BUA analysis, was the main finding present in this study. It was predicted that 70.6% of premenopausal patients could be classified into two groups: osteopenia and osteoporosis. Only 29.45% of the study population was characterized by healthy bone parameters. These data were confirmed by a significant rise in the serum levels of OC, s-BAP, and DPD in the urine of patients with mild and moderate bone loss, indicated by a lower BMD, compared with healthy controls. In addition, bone loss, as measured using BMD and c-BUA, correlated positively with OC, s-BAP, and DPD, and negatively with obesity-related markers such as BMI. Both BMD and ultrasound of the calcaneus (c-BUA), as screening tests for osteoporosis and bone loss and the prediction of hip fractures, have been efficiently evaluated in many studies [47–50]. Additionally, the measurements of urinary DPD and serum OC, and s-BAP, have been proposed as inexpensive bone metabolism markers for the screening of hip and spine osteoporosis [11–15, 51]. Also, previous prospective studies indicate c-BUA as a good predictor of bone fractures among older men and women [52, 53]. Although c-BUA, measured using QUS, could be a pre-screening tool for assessing osteoporosis and may reduce the requirement of using the DXA scan, more research is needed to establish its clinical use [54].

Our study provides suitable combinations of BMD, c-BUA as a static feature of the skeleton, and serum and urine bone biomarkers, such as OC, s-BAP, or DPD, which provide a dynamic measure of the bone remodeling unit as previously reported [55]; these combinations may be helpful in the assessments of osteoporotic risk fractures [56], and especially in the diagnosis of osteoporosis or bone loss in premenopausal patients.

Although the serum biomarkers of bone formation and resorption still have remarkable roles in the diagnosis of bone loss among osteoporotic patients of both genders [11–15, 51], we still have to identify new specific biomarkers that could either alone, or in combination with BMD and c-BUA analyses, provide a better understanding of bone homeostasis or bone loss in these patients. Thus, identifying the signatures of new specific markers, such as miRNAs in osteoporosis, may provide us with more important cell-based information related to bone-loss mechanisms.

Recently, circulating microRNAs have been reported to be associated with many bone diseases [24]. They are short non-coding RNAs which are reported to have regulatory roles in gene expression in most cells and tissues, including muscle and bone [25–27], usually by suppressing translation or destabilizing mRNAs [28].

In this study, we tried to investigate the expression of a set of miRNAs, miR-21, miR-24, mir-100, miR-24a, miR-103-3p, and miR-142-3p, in premenopausal patients with varying degrees of bone loss, indicated by a low BMD, and evaluated its correlation with the scores of BMD, c-BUA, and the biomarkers of bone metabolism. The data showed that miR-21, miR-24, and miR-100 were significantly upregulated and highly expressed in female patients with mild and moderate low BMD compared with controls with normal BMD and healthy bone parameters. Also, the data showed that during osteoporosis, there was a significant downregulation and lower expression of miR-24a, miR-103-3p, and miR-142-3p in association with bone loss status compared to normal cases.

Previous research studies reported on the involvement of mRNAs in the osteoblast genesis process [29–31]. Recently, the upregulation of 49 miRNAs and downregulation of 44 miRNAs were estimated, respectively, during the early to late differentiation stages of osteoclast genesis [32–34]. The interest in miRNAs has come from their associations with physiological or disease conditions, especially regarding their expression and specific signatures in cancer [57]. Thus, miRNAs in the plasma/serum could be used as prognostic circulating biomarkers in many diseases [57–59].

Also, many research studies indicate the potential role of miRNA expression in the function, differentiation, and development of bone in normal and abnormal bone diseases [60–64]. Consistent with our results, previous studies have showed the upregulation of nine miRNAs, including miR-21, miR-24, and miR-100 [65], and downregulation of miR-24a, miR-103-3p, and miR-142-3p [66, 67] in the serum of patients with osteoporosis. The data showed that there was a significant increase in upregulated miRNAs, and that downregulated miRNAs were only significantly decreased in osteoporosis, and that they showed a significant association with BMD. Thus, we can conclude that miR-21, miR-24, and miR-100 are potent inhibitors of bone formation in premenopausal patients with osteoporosis, and miR-24a, miR-103-3p, and miR-142-3p are potent activators of bone formation and are thus significantly increased in healthy bone subjects.

Recently, a circulating miRNAs analysis was performed in patients with osteopenia, osteoporosis, and fragility fractures. In that study, downregulated miR-21 and upregulated mir-133a miRNAs were estimated as potential biomarkers for postmenopausal osteoporosis. These markers showed a moderate to strong correlation with BMD [68]. Although many studies have correlated the expression of miRNAs in the plasma and serum of BMD osteoporotic patients, little is known about the correlation between miRNA expression and c-BUA and serum bone markers.

Thus, our study may be the first to evaluate the correlation between circulating miRNAs c-BUA, OC, s-BAP, and DPD in premenopausal patients with varying degrees of bone loss as measured by lower scores of BMDs. The data showed that the expression of both upregulated miR-21, miR-24, mir-100, and downregulated miR-24a, miR-103-3p, and miR-142-3p correlated positively with bone resorption (DPD) and formation (OC, s-BAP) markers. Similarly, a quantitative estimation of c-BUA and SOS as parameters of BMD in osteoporotic female patients showed a significant correlation with the expressed miRNAs. The values of c-BUA measured using QUS correlated positively with upregulated miR-21, miR-24, and mir-100 and negatively with downregulated miR-24a, miR-103-3p, and miR-142-3p, respectively. The data obtained signify the importance of these miRNAs in osteogenic differentiation in different cell types, which may indicate the homeostatic imbalance between the bone resorption and formation processes that are controlled by osteoclasts and osteoblasts, respectively [60–65].

Based on our results, we propose that miRNAs could have a potential role in the pathogenesis of bone loss. The miRNAs studied were significantly correlated with BMD, c-BUA, and serum bone markers, providing sufficient sensitivity and specificity for distinguishing females with premenopausal osteoporosis.

These new insights into using miRNAs as molecular targets of osteoporosis progression among premenopausal women could be used in the exploration of other related diseases, such as endometrial diseases, and especially precancerous endometrial cancer (EC) lesions involving premenopausal and nulliparous women, or those with pregnancy plans who may prefer more conservative treatment [69]. There are few studies available in the literature that analyze how molecular classification could explain the potential mechanisms related to high EC evolution risk among premenopausal women [70, 71]. It was reported recently that different histopathological or molecular features are present in endometrial diseases with a variety of pathologies [69]. The use of molecular markers has offered the possibility to improve the risk stratification and management of EC [69, 72]. Consequently, detecting and validating the use of different molecular markers like miRNAs in precancerous lesions and associated diseases like premenopausal osteoporosis could change therapeutic strategies, increasing the follow-up of fertility-sparing patients, or tailoring surgical radicality. In addition, molecular markers, such as miRNAs and genomic profiling, might be useful in choosing the most appropriate adjuvant strategies in apparent early-stage EC in pre- and postmenopausal women.

This study has two limitations. Firstly, the recruited bone tissue samples were from female patients; therefore, we were unable to make a sex-comparison analysis of the data. Secondly, although the study is a pilot study with a low sample size, we still need more studies to establish the correlation mechanisms of circulating miRNA biomarkers with high specificity and sensitivity in the pathogenesis of premenopausal osteoporosis.

Nevertheless, our study may be the first to clearly demonstrate the correlation between the profiling of molecular miRNA in premenopausal patients and the scoring rates of bone loss measured using BMD and traditionally used parameters, including c-BUA, OC, s-BAP, and DPD. It also shows that miRNA expression may have potential roles in the detection and classification of osteoporotic diseases.

## Conclusion

A group of circulating miRNAs was shown to be closely correlated with traditionally used parameters, i.e., BMD, c-BUA, OC, s-BAP, and DPD, in bone-health measurement. Additionally, they were identified as non-invasive biomarkers in premenopausal patients with osteoporosis. More studies with large sample sizes are recommended to estimate the mechanistic role of miRNAs in the pathogenesis of osteoporosis and to provide evidence for the use of these miRNAs as a non-invasive method of diagnosing clinical bone loss, especially in premenopausal patients.

## Data Availability

All data generated or analyzed during this study are presented in the manuscript. Please contact the corresponding authors for access to the data presented in this study.
